# Donor Satellite Cell Engraftment is Significantly Augmented When the Host Niche is Preserved and Endogenous Satellite Cells are Incapacitated

**DOI:** 10.1002/stem.1158

**Published:** 2012-06-21

**Authors:** Luisa Boldrin, Alice Neal, Peter S Zammit, Francesco Muntoni, Jennifer E Morgan

**Affiliations:** a The Dubowitz Neuromuscular Centre, UCL Institute of Child HealthLondon, United Kingdom; b Randall Division of Cell and Molecular Biophysics, King's College LondonLondon, United Kingdom

**Keywords:** Niche, Satellite cells, Stem cells, Muscle regeneration, Transplantation

## Abstract

Stem cell transplantation is already in clinical practice for certain genetic diseases and is a promising therapy for dystrophic muscle. We used the *mdx* mouse model of Duchenne muscular dystrophy to investigate the effect of the host satellite cell niche on the contribution of donor muscle stem cells (satellite cells) to muscle regeneration. We found that incapacitation of the host satellite cells and preservation of the muscle niche promote donor satellite cell contribution to muscle regeneration and functional reconstitution of the satellite cell compartment. But, if the host niche is not promptly refilled, or is filled by competent host satellite cells, it becomes nonfunctional and donor engraftment is negligible. Application of this regimen to aged host muscles also promotes efficient regeneration from aged donor satellite cells. In contrast, if the niche is destroyed, yet host satellite cells remain proliferation-competent, donor-derived engraftment is trivial. Thus preservation of the satellite cell niche, concomitant with functional impairment of the majority of satellite cells within dystrophic human muscles, may improve the efficiency of stem cell therapy. Stem Cells*2012;30:1971–1984*

## INTRODUCTION

Stem cells, defined as cells that have the ability to self-renew and commit to a more differentiated state, are present in adult tissues where they mediate tissue maintenance, repair, and regeneration [1]. However, their function may be compromised in aged or diseased tissues. Therefore, transplanting appropriate stem cells derived from normal donors is an attractive proposition to provide long-term treatment of disorders of the hematopoietic system, liver, bone, skin, and skeletal muscle [2–5].

The niche, defined as an “interactive structural unit, organized to facilitate cell-fate decisions in a proper spatiotemporal manner” [6], has a critical effect on stem cells. Although much work has been performed on stem cells in different systems, the interaction between the stem cell and its niche has been little studied. In the most studied stem cell system, the hematopoietic system, emptying of the niche by radiation or chemotherapy allows homing of, and reconstitution by, donor cells and therefore provides a cure for diseases like leukemia [7].

Skeletal muscle is a good model to study the effect of the niche on tissue-specific stem cells. The main skeletal muscle stem cells, satellite cells, are located underneath the basal lamina of the muscle fibers [8]. Thus the satellite cell niche is described as the anatomical location between the myofiber and its basal lamina; the presence of a satellite cell is required in order for the niche to be functional [9]. In vitro work on isolated myofibers shows that the fiber and its basal lamina are the cellular and extracellular matrix components required and sufficient for satellite cell activation, proliferation, migration, and differentiation [10, 11].

In response to muscle injury, satellite cells activate, proliferate, and then either repair or replace damaged muscle fibers, or self-renew [12]. Recent work has shown that satellite cells, rather than other stem cells, are the source of muscle regeneration [13–15].

The regenerative process is highly dependent on the age or pathological status of the host environment [16, 17]. For example, in Duchenne muscular dystrophy (DMD), muscle fibers undergo necrosis due to dystrophin deficiency. At first, endogenous satellite cells repair damaged muscle fibers, but fiber fragility causes chronic degeneration. Cycles of regeneration-degeneration continue until regeneration fails to keep up and muscle becomes wasted and replaced by fibrous/adipose/connective tissue [18, 19].

The *mdx* mouse is a naturally occurring genetic and biochemical homolog of DMD that has been extensively used as a model of skeletal muscle regeneration. Recently, *mdx* mice with shortened telomeres were shown to have exacerbated muscle pathology, highlighting the role of satellite cells in muscular dystrophies [20].

As young adult *mdx* muscles regenerate well, the *mdx* mouse is not a perfect model of DMD. In an attempt to exacerbate the muscle pathology to make it more similar to DMD, high doses of radiation have been applied locally [21–23]. This treatment preserves the postmitotic muscle fibers but reduces the growth of the muscle and other surrounding tissues (e.g., bones). The majority of satellite cells are radiation-sensitive and die, leading to a severe reduction in regeneration of irradiated dystrophic *mdx* skeletal muscles. However, a minority of satellite cells survives radiation and can be recruited to muscle regeneration [24]. These cells, which are rare within skeletal muscles of normal mice, are even rarer in *mdx* mice [24, 25].

The type and extent of skeletal muscle injury determine its regenerative response [26]. Several muscle injury models have been used to study muscle regeneration, including examining the dynamics of the activation, proliferation, and differentiation of muscle stem cells in normal and dystrophic rodents [27, 28]. These injuries have different effects on skeletal muscle. Cryoinjury destroys cells local to the injury site but preserves the basal lamina of muscle fibers [29]. Following cryoinjury, skeletal muscle is capable of regeneration, indicating that at least some satellite cells either survive the injury, or move into damaged areas from elsewhere within the muscle, or from neighboring muscles. Injection of myotoxins, such as notexin, cardiotoxin, or barium chloride, destroys muscle fibers but preserves the muscle fiber basal lamina, nerves, blood vessels, and satellite cells [27].

We performed experiments designed to test the effect of the host satellite cell niche on donor satellite cell engraftment. We made a rigorous, systematic study of the regenerative capacity of donor mouse satellite cells grafted into host mouse skeletal muscles that had been modulated in different ways (cardiotoxin, notexin, barium chloride [27], cryoinjury [29], and irradiation [24, 30]), which either destroy or preserve the niche. To test donor satellite cell regeneration and self-renewal, we used a controlled experimental model, in which the genetic background of the host and donor mice, the donor cell preparations, and the time points studied were constant. We provide clear evidence that modulation of the host satellite cell niche is critical for efficient donor satellite cell engraftment.

## MATERIALS AND METHODS

### Host Mice and Muscle Injury

Breeding of mice and experimental procedures were carried out in the Biological Services Unit, UCL, in accordance with the Animals (Scientific Procedures) Act 1986. Three-week-old *mdx* nude mice [31] were anesthetized with isoflurane or hypnorm and hypnovel and *tibialis anterior* (TA) muscles were injured in one of the following ways: 18 Gy radiation at a dose rate of 0.72 Gy/minute administered to the hind limbs [30] or injection into TA muscles of one of the following: 25 μl of 1.2% barium chloride (BaCl_2_) (Sigma, Gillingham,U.K., http://www.sigmaaldrich.com)) [32], 10 μl of *Notechis scutatus* notexin (10 μg/ml) (Latoxan, Valence, France, http://www.latoxan.com) [25, 33], and 50 μl of 10 μM cardiotoxin (Latoxan, Valence, France, http://www.latoxan.com) [34]. Vetergesic was administered as analgesic after all treatments except irradiation.

### Histological Analyses of Injured Muscles

Three days or 4 weeks after irradiation, myotoxin, or no treatment, muscles were frozen in isopentane chilled in liquid nitrogen. Representative serial transverse 7-μm cryosections were stained with either hematoxylin and eosin (H&E), acid phosphatase (AP) (protocol published in [35]), or an antibody to CD45 (AbD Serotec, Kidlington, U.K., http://www.abdserotec.com)—in order to detect inflammatory cells—or were immunostained with antibodies to CD31 (Abcam, Cambridge, U.K., http://www.abcam.com) to identify endothelial cells, or Pax7 (Developmental Studies Hybridoma Bank (DSHB), Iowa City, Iowa, U.S.A., http://dshb.biology.uiowa.edu/) and laminin (Sigma-Aldrich, Gillingham, U.K., http://www.sigmaaldrich.com) to identify satellite cells under the basal lamina of muscle fibers. Nuclei were counterstained with 4′,6-diamidino-2-phenylindole (DAPI). Numbers of satellite cells per muscle fiber in representative transverse sections were counted. The percentage of the area of the muscle occupied by inflammatory infiltrate, or endothelial cells, on representative transverse sections was estimated by ImageJ software (http://rsbweb.nih.gov/ij/).

### Cytokine Quantification

Proteins were extracted from snap-frozen TA muscles of 3-week-old mice that were either non-treated or had been irradiated 3 days before or on the same day as muscle analysis. Right and left TAs of the same mouse treated in the same way were pooled and cytokines quantified by Quantibody Mouse Cytokine Array (RayBiotech, Inc., Norcross, GA, U.S.A., http://www.raybiotech.com). Results were analyzed and visualized in heat map using Cluster and TreeView softwares (http://rana.lbl.gov/EisenSoftware.htm).

### Donor Satellite Cell Preparation

Young adult (1–5 months old) *3F-nlacZ-2E* or *Myf5^nlacZ/+^* genetically modified mice were used as donors: β-galactosidase (β-gal) is expressed in myonuclei in 3F-*nlacZ*-2E mice and in satellite cells in the *Myf5^nlacZ/+^* strain. This allows us to identify myonuclei or satellite cells of donor origin within grafted muscles. *Extensor digitorum longus* (EDL) muscle fibers were isolated as previously described [36] and approximately 400 satellite cells physically stripped from these fibers [11, 37] were grafted into each host TA muscle, as previously described [37]. Muscles were removed for analysis 4 weeks after grafting. In the experiment where donor satellite cells were irradiated prior to engraftment, donor mice had both hind limbs irradiated at 4.5, 9, 18, or 25 Gy, 3 days before satellite cells were prepared from their EDL muscles.

### Satellite Cell Grafting into Host Muscles Modulated by Different Injury Regimens

To determine the effect of the different injuries to host muscle on donor satellite cell engraftment, donor satellite cells were grafted into TA muscles of 3-week-old host *mdx* nude mice. TA muscles were injured (as described above) either at the time of grafting or 1, 2, or 3 days beforehand. We also tested cryoinjury [34, 38–40] as a muscle pretreatment, but as this is an invasive and moderately severe procedure, it was performed only at the time of cell injection [38]. Non-treated littermate control mice were grafted with the same donor cells in each experiment. We also tested the combination of irradiation and notexin treatment of host muscle on donor satellite cell engraftment.

*Mdx* nude mice had their hind limbs irradiated. In some mice, notexin (10 μl of 10 μg/ml) was injected into the TA muscles of irradiated limbs either immediately after irradiation or at the time of grafting. Cells were grafted either 3 days or 4 weeks after irradiation (Fig. 6A).

To explore the effect of different doses of radiation on donor satellite cell engraftment, both hind limbs of *mdx* nude mice were irradiated at 4.5, 9, 18, and 25 Gy, 3 days before grafting. We also investigated grafting satellite cells into host muscles that had been given either 18 or 25 Gy immediately before grafting.

### Aged Satellite Cell Grafting into Young and Aged Host Mouse Muscles

To investigate the effect of irradiation on donor-derived muscle regeneration and self-renewal in aged muscle tissue, satellite cells were isolated from aged (1.5-year old) donor mice and grafted into TA muscles of both 3 weeks and 9-month-old host *mdx* nude mice that had their hind limbs irradiated 3 days before grafting [37].

To determine functionality of aged donor cells grafted into aged dystrophic muscle, 10 μl notexin (10 μg/ml) was injected 3 weeks after cell grafts, to cause muscle fiber destruction but preserve satellite cells as well as muscle fiber basal lamina and microcirculation [27]. A week after myotoxin injection, muscles were collected for cryosectioning (Fig. 4A) [25].

### Analyses of Grafted Muscles

Seven micrometers serial transverse cryosections were cut at intervals of 100 μm throughout the entire muscles that had been grafted with 3F-*nlacZ*-2E donor cells. Sections were stained with X-gal and serial sections to those containing the most myonuclei of donor origin were stained with P7 dystrophin antibody and counterstained with DAPI, as described previously [37, 41]. The presence of *lacZ* indicates that particular group of fibers is of donor origin, rather than being host revertant fibers; as 3F-*nlacZ*-2E is a nuclear marker that becomes silenced following cell proliferation, we use dystrophin expression instead for quantifying fibers of donor origin. Counts were made of the number of donor (dystrophin positive) fibers in transverse sections serial to those that contained most donor X-gal-positive myonuclei. INSTAT software was used for statistical analysis.

Muscles that had been grafted with *Myf5^nlacZ/+^* satellite cells were removed 4 weeks after grafting, fixed in paraformaldehyde, and X-gal stained as described previously [41]. Cells expressing β-Gal were satellite cells of donor origin.

In the experiment where muscles grafted with *Myf5^nlacZ/+^* satellite cells were notexin-injured (Fig. 4A), muscles were frozen and 7 μm serial transverse cryosections were cut at intervals of 100 μm throughout the entire muscles. Sections were double-stained with X-gal and laminin antibody, in order to identify donor-derived satellite cells. Sections serial to those containing the highest number of X-gal-positive nuclei were immunostained with P7 dystrophin [42] and BF-34 neonatal myosin antibody (DSHB). Counts were made of donor-derived satellite cells either localized immediately underneath (satellite cells) or outside (myoblasts) the basal lamina, or centrally located within muscle fibers (newly regenerated myonuclei). We make this distinction because the *Myf5* locus is active not only in satellite cells but also in myonuclei in recently regenerated muscle fibers [43]. In serial sections, the number of donor-derived dystrophin and neonatal myosin-positive fibers was counted.

### Single-Fiber Immunohistochemistry

Single fibers isolated from irradiated (18 or 25 Gy) EDL muscles of 3–4-week-old *mdx* nude and C57Bl/6 mice were fixed in 4% paraformaldehyde, permeabilized with 0.5% Triton X-100 (Sigma), and blocked with 10% goat serum. Fibers were incubated overnight at 4°C with the primary antibody Pax7 (DSHB). After washing in phosphate buffered saline (PBS), and incubation with the appropriate Alexa-Fluor secondary antibody, fibers were individually placed onto glass microscope slides and analyzed.

### Statistical Analysis

Results are expressed as mean ± SEM from an appropriate number of samples as reported in the figure legends. Statistical significance was determined by Student's *t* test.

### Microscopy

Fluorescence and bright-field microscopy images were captured using a Zeiss Axiophot (Carl Zeiss, Cambridge, U.K., http://www.zeiss.com) microscope and Metamorph (MetaMorph Software, Sunnyvale, California, U.S.A., http://www.moleculardevices.com) software. Macroscopic pictures of whole X-gal-stained muscles were captured with a Leica stereomicroscope (Leica, Bucks, U.K., http://www.leica.com). Minor adjustments to the quality of images were done using Adobe Photoshop CS2 (Adobe Photoshop, Berkshire, U.K., http://www.Adobe.com).

## RESULTS

### The Effect of Different Modulatory Regimens on the Host Environment

**Figure 1:**
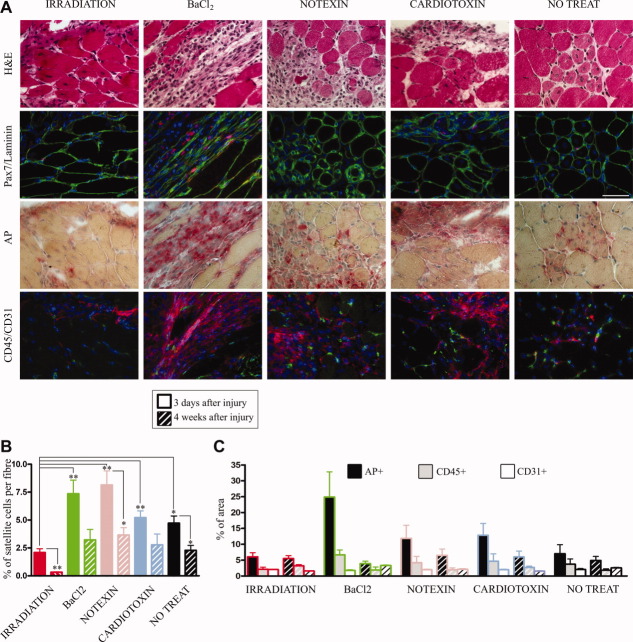
Satellite cell number and inflammation within mdx nude muscles injured in different ways. **(A–C):** Serial transverse cryosections of irradiated, BaCl_2_, notexin, or cardiotoxin treated or nontreated (no treat) *mdx* nude *tibialis anterior* muscles were stained with H&E, Pax7, laminin antibodies, AP, CD45, and CD31 antibodies at 3 days (*n* = 4 in each group) and 4 weeks after treatment (*n* = 4 in each group, except for BaCl_2_ where *n* = 2). (A): Representative images of muscle histology 3 days after injury. Immunostaining for Pax7 and laminin revealed satellite cells (red) below the basal lamina (green). Scarce inflammatory (red AP+ and red CD45+) cells were present in irradiated muscles, whereas inflammation was more evident in muscles injured by all myotoxins. In nontreated muscles, sporadic foci of inflammation were found. Similar endothelial CD31+ area (green) was detected between control, myotoxin-treated, and irradiated muscles. Graphs depict percentage of satellite cell number (B) and inflammatory area (C) in muscles treated 3 days and 4 weeks previously, compared to control, nontreated muscles of the same ages. Satellite cell number per fiber was significantly lower in irradiated compared to all muscles treated in other ways (*, *p* < .05; **, *p* < .01). The number of satellite cells per fiber was significantly reduced 4 weeks compared to 3 days after irradiation and in aged-matched nontreated mice. Number of satellite cells per fiber returned to control levels 4 weeks after myotoxin treatment (B). There was no significant difference in the percentage of AP+ or CD45+, or CD31+ area between control, myotoxin-treated, and irradiated muscles (C). Scale bar = 25 μm.

We wished to investigate the effect of different modulatory regimens (18 Gy irradiation and myotoxin-induced injuries) on satellite cell number and inflammatory/endothelial cell area within host *mdx* nude mouse muscles. We found, on sections of TA muscles (Fig. 1A), that satellite cell number per fiber was significantly decreased 3 days after irradiation (2 ± 0.3), compared to both myotoxin-injured (7.4 ± 1.2 in BaCl_2_; 8.1 ± 1.3 in notexin; 5.2 ± 0.6 in cardiotoxin-treated muscles) and nontreated muscles (4.7 ± 0.6) (Fig. 1B). The number of satellite cells per fiber was even further reduced 4 weeks (0.3 ± 0.0) after irradiation (Fig. 1B). In contrast, we found no significant difference in the number of satellite cells between myotoxin-treated and -nontreated muscles (Fig. 1B). These data show that host satellite cell number is significantly reduced in irradiated compared to myotoxin-treated and nontreated muscles, evidence that, in irradiated muscles, fewer host satellite cells can be recruited for muscle regeneration. However, the basal lamina survives after irradiation or injection of myotoxins and at least some satellite cells remain present within their niche on the myofiber (Fig. 1).

In *mdx* nude mouse muscles injected with myotoxins, muscle fiber necrosis and inflammatory infiltrate were evident 3 days after treatment (Fig. 1A). Inflammatory cells were localized both in the affected area of treated and in nontreated muscles, but their presence was less evident in irradiated muscles (Fig. 1A, 1C). Although there was no significant difference in the area of inflammation present in any of the treated muscles compared to nontreated muscles at either 3 days or 4 weeks after injury, it is obvious that fewer inflammatory cells were present 3 days after irradiation compared to myotoxin-treated muscles (Fig. 1C). We found no significant difference in endothelial area between control muscles, myotoxin-treated muscles, and those irradiated with 18 or 25 Gy (supporting information Fig. S1), indicating that neither irradiation nor myotoxin treatment had an obvious detrimental effect on blood vessels.

### Incapacitation of the Host Satellite Cells and Preservation of the Niche Promotes Robust Donor Satellite Cell-Mediated Engraftment

**Figure 2:**
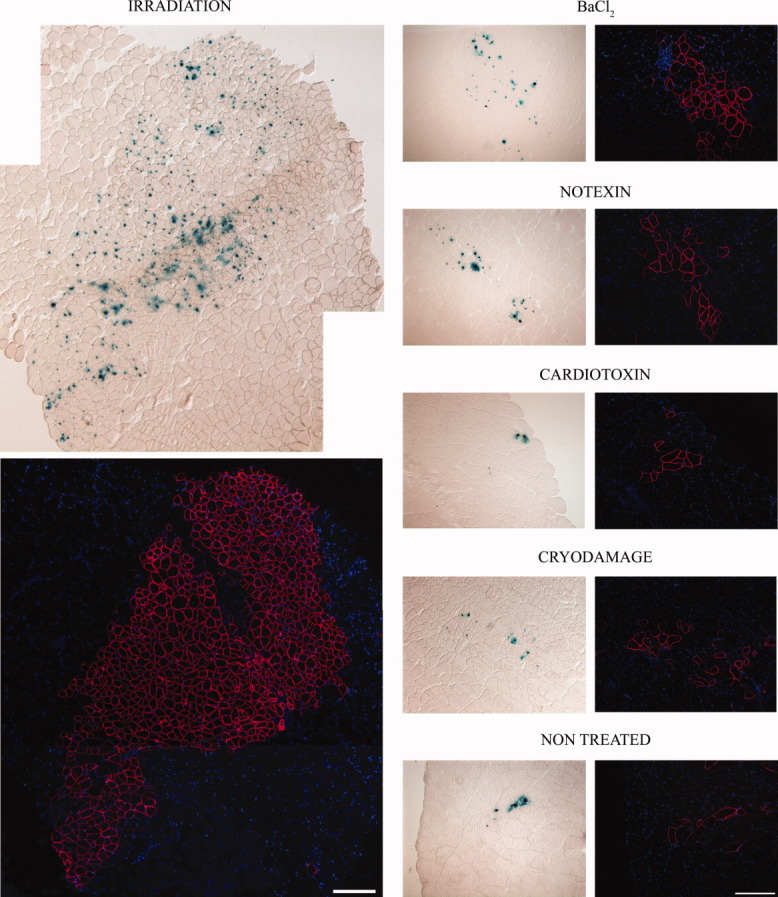
Muscle regeneration mediated by 3F-nlacZ-2E satellite cell grafts in host muscles injured in different ways. *Mdx* nude *tibialis anterior* muscles were irradiated, or injured, or nontreated, before being grafted 3 days later with 3F-*nlacZ*-2E satellite cells and removed for analysis after a further 4 weeks. Representative pictures of X-gal stained and dystrophin immunostained serial transversal sections (from grafts detailed in Table 1) are presented for each of the muscle injuries that were tested: X-gal-positive myonuclei localized inside dystrophin-positive fibers show donor-derived muscle fibers. The amount of muscle formed by donor satellite cells is clearly higher in the irradiated host muscles than in muscles injured by the other means, which only contain small groups of dystrophin-positive fibers. Nuclei are counterstained with DAPI. Scale bar = 100 μm.

It is evident that donor satellite cells robustly contribute to muscle regeneration within host muscles that had been preirradiated [37, 41]. However, it is not known whether other pretreatment regimens of host muscle induce better muscle regeneration from donor cells. We therefore compared the effect of treating dystrophic immunodeficient host muscles by irradiation or different injury regimens either at the time of, or up to 3 days before, grafting donor satellite cells. Significantly, more muscle fibers of donor origin were found in host muscles that had been irradiated with 18 Gy either 3 (589 ± 149) (*p* < .001), 2 (418 ± 83) (*p* < .001), or 1 day (237 ± 54) (*p* < .01) prior to and on the same day (433 ± 75) (*p* < .0001) as grafting, than in nontreated muscles (11 ± 4) (Table 1). In all the other muscle injuries tested—notexin, cardiotoxin, and cryodamage—no significant difference was found in the amount of donor-derived muscle regeneration compared to nontreated muscles (Table 1 and Fig. 2). Only when BaCl_2_ was injected 3 days before cell grafting, was the amount of donor-derived muscle significantly higher (51 ± 15) (*p* < .05) compared to the nontreated group (Table 1). However, the amount of muscle formed in these grafts was significantly less than in muscles irradiated at any time point before grafting (*p* < .05) (Table 1). Notably, using our direct comparison experiments, we obtained at least 50 times more muscle of donor origin in irradiated compared to nontreated host muscles and at least 10 times more than in barium chloride-treated host muscles. No significant difference was found in the amount of donor-derived muscle when cells were grafted 2 days (35 ± 15) or 1 day after (6 ± 3), or on the same day (7 ± 3) as BaCl_2_ injection. We therefore conclude that donor satellite cells robustly contribute to muscle regeneration in preirradiated muscles.

**Table 1:** Donor-derived muscle formation in response to different injury regimes

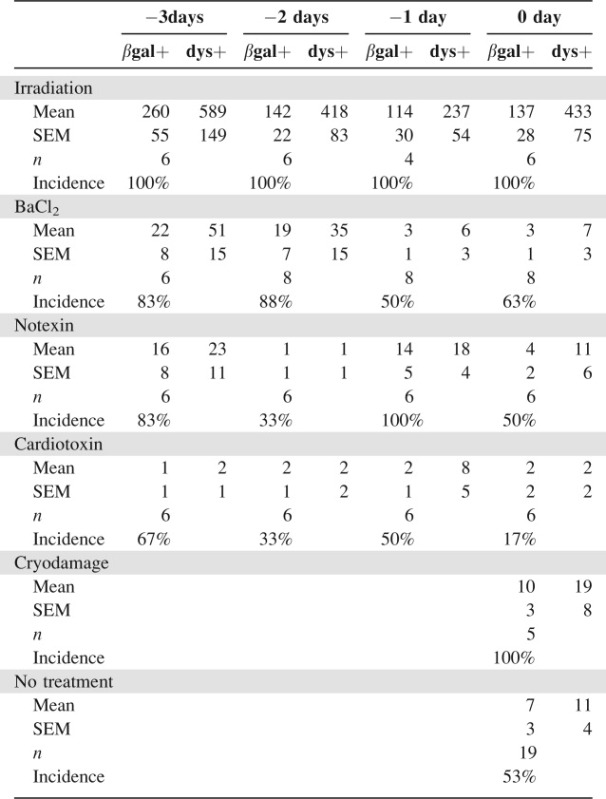

It is possible that, even if donor satellite cells do not give rise to regenerated muscle fibers in a particular host muscle environment, they may still self-renew. We therefore investigated donor-derived satellite cell self-renewal in muscles modulated by the different pretreatment regimens. More satellite cells of donor origin were clearly present within host muscles that had been irradiated, compared to BaCl_2_, cardiotoxin, notexin, or nontreated muscles (supporting information Fig. S2). We therefore conclude that self-renewal and muscle regeneration of donor satellite cells are linked and occur efficiently only when the host muscle has been preirradiated (supporting information Fig. S2).

### Cytokine Expression in Irradiated Compared to Non Irradiated Host Muscles

There is evidence that dystrophic muscles have dysregulated expression of some cytokines [44–46], and irradiation, albeit at different doses and in different tissues, has been shown to alter cytokine expression [47–49]. We therefore used a commercially available array to investigate changes in cytokine expression in *mdx* nude muscles that were irradiated either 3 days before, or on the same day as, analysis, since the effect of irradiation at both these time points promotes similar donor-derived muscle regeneration (Table 1). However, we found cytokine expression in TA muscles to be variable between males and females (supporting information Fig. S3). Among the pool of cytokines on the array, 25 were not expressed in skeletal muscle and none of the remaining 95 was differentially expressed in the same direction at both time points in both males and females. We therefore conclude that changes in levels of these cytokines within host dystrophic muscles are not crucial modifiers of donor satellite cell function.

### Modulation of the Aged Host Environment Promotes Aged Donor Satellite Cells to Regenerate and Self-Renew Effectively

We have already shown that old donor satellite cells can regenerate well in a young host environment, and young cells in an old environment, when the host muscle had been modulated by radiation [37, 50]. But it remains possible that aged satellite cells are unable to mount a robust regenerative response in an aged dystrophic host environment, in which muscles are wasted and fibrotic [37]. We therefore investigated whether satellite cells from aged donors could regenerate as well in old *mdx* nude host mice as they do in young host mice.

**Figure 3:**
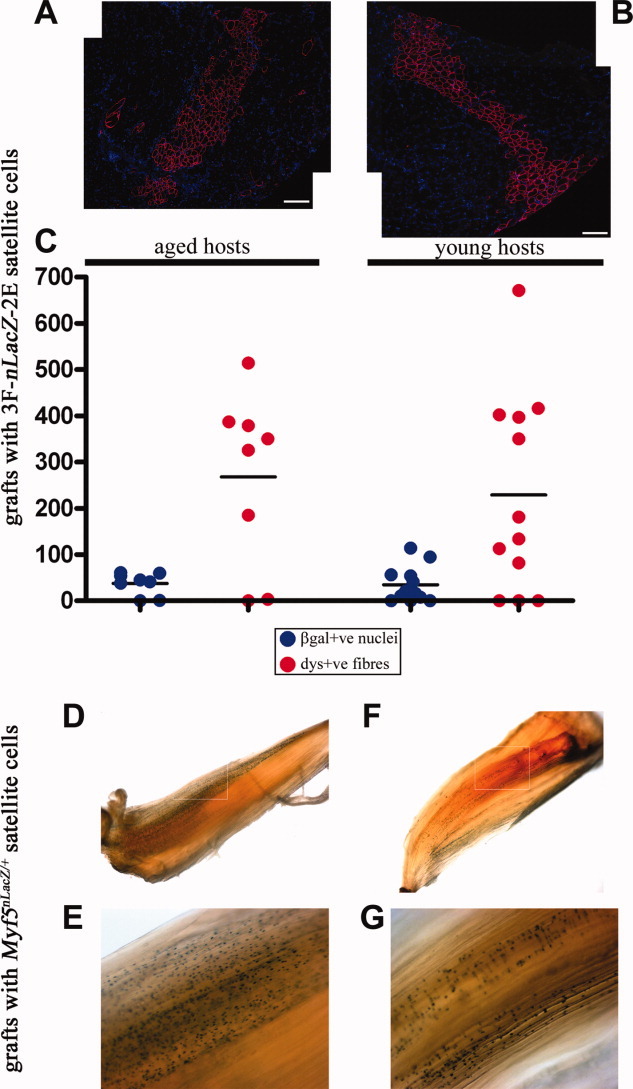
Muscle regeneration and self-renewal mediated by aged 3F-nlacZ-2E and My5^nlacZ/+^ donor satellite cells in irradiated muscles of aged *mdx* nude hosts. Satellite cells, isolated from 3F-*nlacZ*-2E donor mice (*n* = 3), formed a similar amount of donor-derived fibers in aged (*n* = 8) **(A)** as in young (*n* = 12) **(B)** host mice. Scale bar = 100 μm. Nuclei are counterstained with DAPI. **(C):** Depicts the amount of dystrophin-positive fibers and donor-derived myonuclei in individual grafts. No significant difference was detected either in the amount of donor-derived muscle, or in the incidence of engraftment, between young and aged grafted muscles. When grafted satellite cells were isolated from *My5^nlacZ/+^* aged donor mice (*n* = 2), their self-renewal potential was evaluated in aged (*n* = 6) and young (*n* = 8) host mice. Whole X-gal-stained grafted muscles showed the presence of β-gal expressing satellite cells along the fibers in aged **(D)** and young **(F)** host *mdx* nude mice. Donor-derived satellite cell distribution and amount was similar in both aged **(E)** and young **(G)** host muscles and the incidence of positive engraftments was high in both cases (75% and 100%, respectively). Magnifications: ×2 and ×10.

Surprisingly, aged satellite cells were able to contribute to muscle regeneration to a similar robust extent in aged as in young irradiated host muscles (Fig. 3A, 3B). There was no significant difference in the number of donor fibers generated in aged (268 ± 66) compared to young (229 ± 62) recipient mice (Fig. 3C). Similarly, aged *Myf5^nlacZ^*^/+^ donor satellite cells gave rise to donor-derived nuclei equally well in aged (Fig. 3D, 3E) as in young mice (Fig. 3F, 3G). Thus, radiation modulates the aged muscle environment such that it restores its ability to support efficient regeneration.

**Figure 4:**
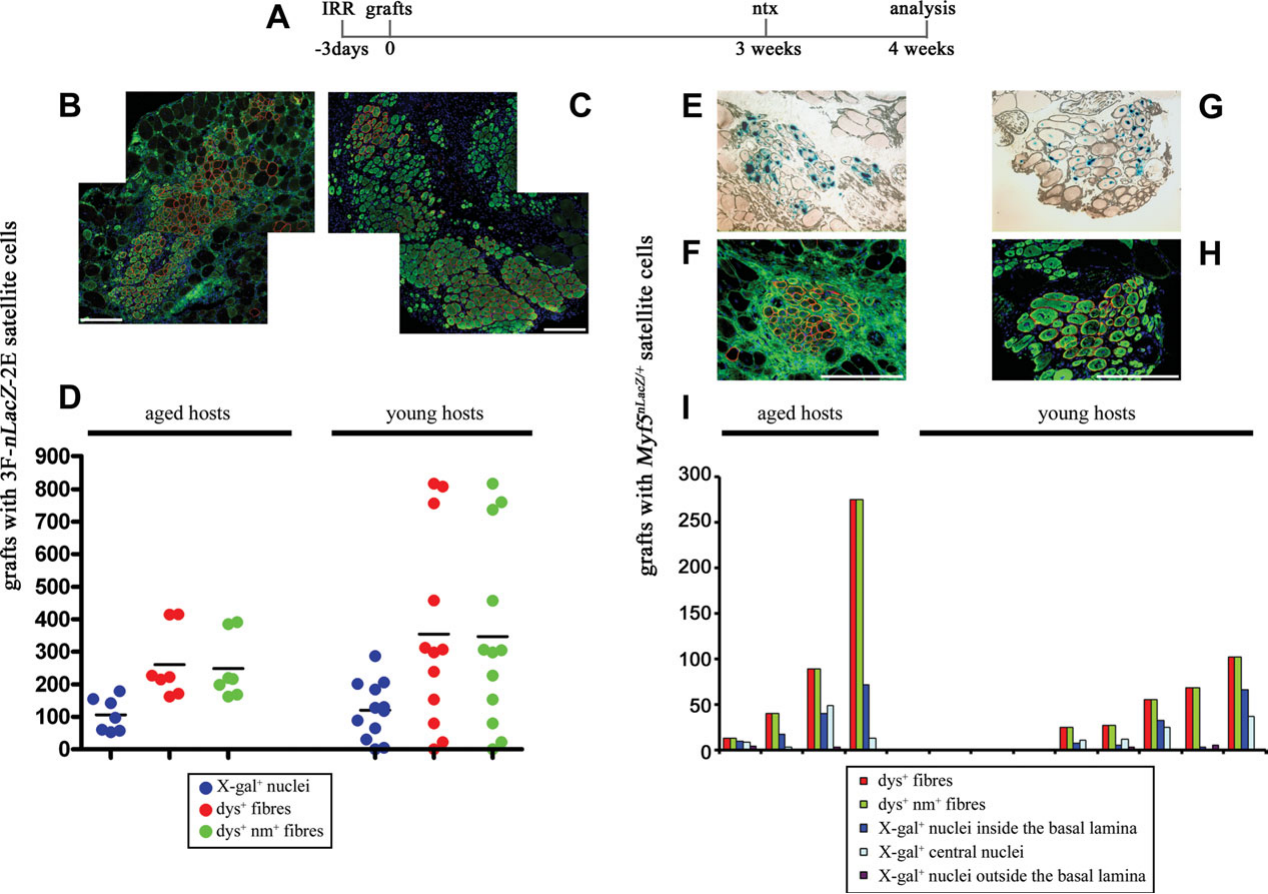
Functionality of aged 3F-nlacZ-2E and My5^nlacZ/+^ donor satellite cells grafted in aged *mdx* nude muscles. **(A):** Schematic representation of the experimental design to assess functionality of grafted aged satellite cells: hind limbs of recipient mice were irradiated (IRR) 3 days before cell grafts, TA muscles were injured with notexin 3 weeks later and analyzed a week later. When satellite cells were isolated from 3F-*nlacZ*-2E mice (*n* = 5), donor-derived muscle fibers were detected in grafted muscles in transverse cryosections by immunostaining for dystrophin (red) and neonatal myosin (green), indicating the fibers formed by donor satellite cells were newly regenerated, both in aged (*n* = 7) **(B)** and young (*n* = 12) **(C)** host muscles. No significant difference was detected in the number of donor-derived fibers between young and aged host muscles **(D)**. Donor satellite cells, derived from 1.5-year-old *Myf5^nlacZ^*^/+^ mice (*n* = 3), were identified on transverse sections of grafted muscles by X-gal staining and their location below the basal lamina was identified by laminin immunostaining, both in aged (*n* = 4) **(E)** and young (*n* = 8) **(G)** recipient muscles. Fibers containing donor-derived satellite cells were also dystrophin and neonatal myosin positive, both in aged **(F)** and young **(H)***mdx* nude mice. Quantification of donor-derived fibers and cells in each graft is presented in detail in the graph **(I)**. Scale bar = 100 μm.

Next, we assayed the functionality of satellite cells derived from aged donor mice within aged dystrophic host muscles (Fig. 4A). Challenge of grafted, regenerated muscles with notexin is an assay of the functionality of muscle stem/precursor cells of donor origin [24, 25, 37, 50–54]: the finding of newly regenerated muscle fibers of donor origin 7 days after notexin injection is evidence that some of the grafted cells had given rise to functional muscle stem cells, which are able to contribute to muscle regeneration after muscle injury.

Newly regenerated fibers of donor origin were present in similar numbers in aged (248 ± 47) (Fig. 4B) and young (347 ± 83) (Fig. 4C) host muscles (Fig. 4D), indicating that the grafted cells had given rise to functional satellite cells. To confirm that the donor cells had indeed reconstituted the satellite cell niche, we repeated our functional assay using *Myf5^nlacZ^*^/+^ donor cells. We found donor-derived nuclei expressing *Myf5^nlacZ^*^/+^ in similar numbers after subsequent injury in both aged (Fig. 4E, 4F) and young (Fig. 4G, 4H) grafted muscles. The majority of donor *Myf5^nlacZ^*^/+^ cells were immediately underneath the basal lamina of newly regenerated muscle fibers, indicating that they were satellite cells. The number of donor-derived satellite cells in aged (38 ± 16) and young (14 ± 7) hosts that were below the basal lamina of newly regenerated muscle fibers (104 ± 59 in aged and 35 ± 13 in young hosts) was not significantly different (Fig. 4I). This is evidence that aged-grafted donor cells had given rise to functional progeny in both young and aged environments.

### Satellite Cells Are Heterogeneous on the Basis of Their Sensitivity to a DNA-Damaging Reagent

**Figure 5:**
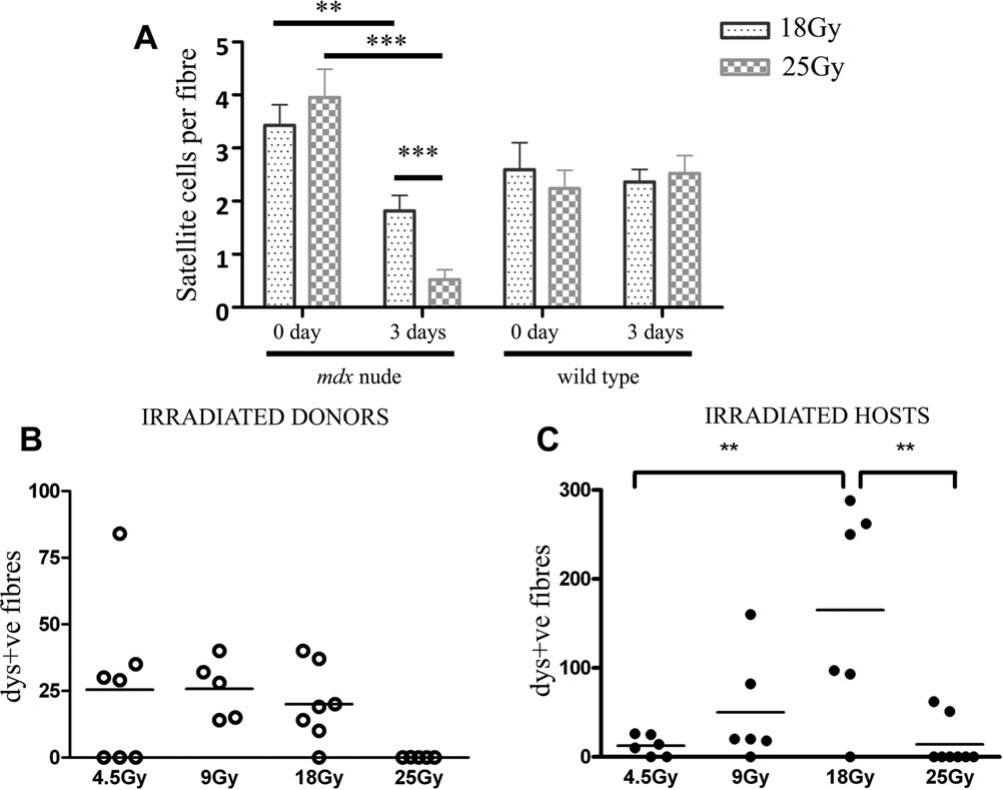
The effect of different doses of radiation on satellite cells and engraftment efficiency. *Mdx* nude and wild-type C57Bl/6 mice whose hind legs were irradiated with 18 Gy (*n* = 2 and 4, respectively) or 25 Gy (*n* = 2 and 4, respectively) had both *Extensor digitorum longus* (EDL) muscles removed either on the same day as irradiation (day 0) (*n* = 1 and 2, respectively) or 3 days later (*n* = 1 and 2, respectively). The number of Pax7+ve satellite cells per fiber, counted on at least 20 EDL myofibers per mouse, is depicted in the graph as mean ± SEM **(A)**. The number of satellite cell per EDL fiber was significantly reduced in *mdx* nude mice 3 days after 18 or 25 Gy irradiation. Fewer satellite cells survived 3 days after 25 Gy compared to 18 Gy irradiation (***, *p* < .001). **(B):** Satellite cells isolated from donor mice whose hind limbs had been irradiated 3 days previously with 4.5, 9, 18, and 25 Gy (*n* = 2 per each radiation dose) were grafted into preirradiated (18 Gy) host TA muscles (*n* = 7, 5, 7, and 5, respectively, for 4.5, 9, 18, and 25 Gy irradiated cells). The number of donor-derived dystrophin-positive fibers obtained in each graft is depicted. **(C):** Graph depicts the number of donor fibers in host *tibialis anterior* (TA) muscles irradiated with 4.5 (*n* = 6), 9 (*n* = 6), 18 (*n* = 6), and 25 (*n* = 8) Gy and grafted 3 days later with satellite cells freshly isolated from donor mice (*n* = 4). The amount of donor-derived muscle was significantly higher in host muscles irradiated with 18 Gy than in muscles irradiated with 25 and 4.5 Gy (**, *p* < .01).

Satellite cells are known to be heterogeneous [55–59]. As stem cells in different systems are resistant to cytotoxins or radiation [60–62], we hypothesized that resistance to radiation would reveal the more stem cell-like satellite cells. By applying different radiation doses to normal donor 3F-*nlacZ*-2E mouse hind limbs 3 days before preparing satellite cells for grafting, we show that, although satellite cells in normal mice are not destroyed 3 days after receiving 25 Gy (Fig. 5A), this radiation dose completely obliterates their regenerative capacity (Fig. 5B). However, at least some satellite cells within these normal donor mice are resistant to 4.5, 9, or 18 Gy and contribute to small, yet conspicuous, amounts of muscle regeneration (25 ± 11, 26 ± 5, 20 ± 5 donor-derived muscle fibers, respectively) within host *mdx* nude muscles that had been irradiated with 18 Gy (Fig. 5B). Therefore, radiation-resistant satellite cells within normal mouse muscles are capable of contributing to regenerated muscle fibers.

If host muscles are preirradiated at lower doses (4.5 or 9 Gy), donor satellite cell engraftment (derived from nonirradiated donor muscles) is reduced (13 ± 5 and 50 ± 25 donor fibers, respectively) (Fig. 5C). Since some satellite cells are resistant to these doses of radiation (Fig. 5B) [24, 30], functional host satellite cells can outcompete donor cells, thus reducing the efficiency of donor cell engraftment (Fig. 5C). Surprisingly, ablation of host satellite cells allows almost no donor cell engraftment: in fact, as little muscle of donor origin was formed when host muscles were irradiated with 25 Gy—which had no obvious detrimental effect to the host muscle fibers or structure (supporting information Fig. S1)—(14 ± 9 donor fibers), as in nonirradiated host muscles (11 ± 4) (Fig. 5C and Table 1).

### The Status of the Host Satellite Cell Niche Is Vital for Efficient Engraftment

**Figure 6:**
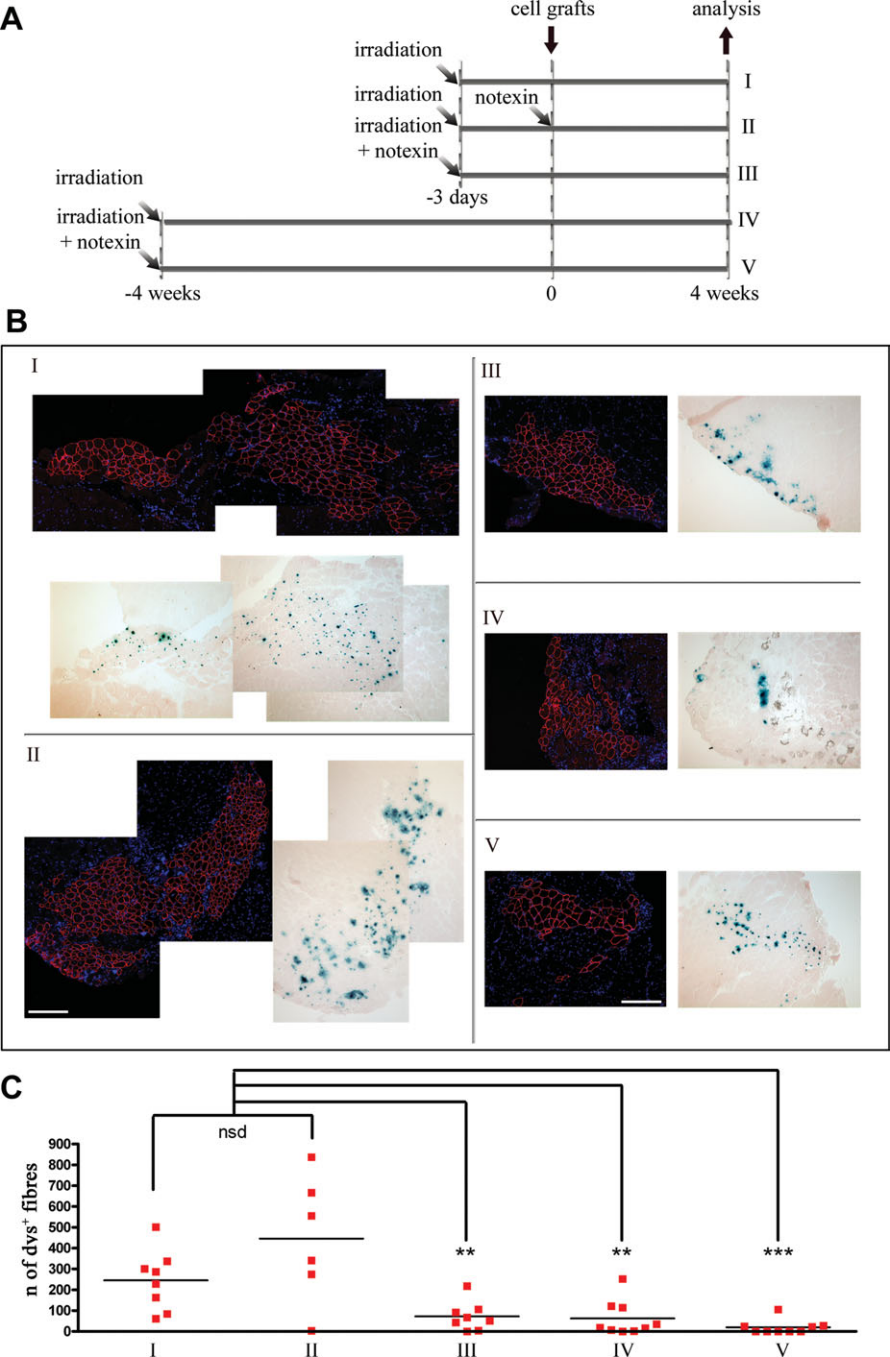
Donor-derived muscle formation after treatment of host muscle with irradiation and notexin. **(A):** Experimental design. Satellite cells, freshly isolated from 3F-*nLacZ*-2E donor mice (*n* = 6), were grafted in *tibialis anterior* (TA) muscles of 3-week-old *mdx* nude mice pretreated in different ways and removed 4 weeks after engraftment. Muscles had been irradiated either 3 days (I) (*n* = 8) or 4 weeks (IV) (*n* = 9) before cell grafting. Some muscles irradiated 3 days beforehand were also injected with notexin, either immediately after irradiation (III) (*n* = 8) or at the time of cell injection (II) (*n* = 6). Some of the muscles irradiated 4 weeks before cell grafts also had their TA muscles injected with notexin immediately after irradiation (V) (*n* = 9). **(B):** Transverse serial sections of engrafted muscles were X-gal stained and immunostained with dystrophin antibody to detect donor-derived fibers. Scale bar = 100 μm. **(C):** Quantification of dystrophin-positive fibers in each graft is given in the graph. No significant difference was found in the amount of donor-derived muscle between muscles grafted as in (I) and (II) that had significantly more donor-derived fibers than in all the other grafted muscles (III--V) (**, *p* < .01; ***, *p* < .001).

We have shown that the satellite cell pool in *mdx* nude muscles was further reduced between 3 days and 4 weeks after 18 Gy irradiation (Fig. 1C and supporting information Fig. S4). We then stimulated irradiated muscles by notexin and showed that radiation-resistant satellite cells were able to partially refill the niche (2.4 ± 0.4) (supporting information Fig. S4). Next, we determined whether donor-derived muscle regeneration is enhanced when satellite cells are severely depleted. We investigated whether radiation-resistant host satellite cells activated by notexin either at the time of, or before, cell grafts were able to compete with donor satellite cells, as described in Figure 6A. Interestingly, the number of donor-derived fibers was similar between muscles grafted 3 days after irradiation (245 ± 51) and muscles injected with both cells and notexin 3 days after irradiation (446 ± 122) (Fig. 6B-I, 6B-II, 6C), indicating that just activated endogenous radiation-resistant satellite cells did not compete with donor cells. However, if endogenous radiation-resistant satellite cells were activated 3 days before cell grafts, the number of donor-derived fibers was significantly lower (73 ± 25) (Fig. 6B-III, 6C), implying that radiation-resistant satellite cells that were already in an activated state outcompeted exogenous satellite cells. A similar significantly lower amount of donor-derived fibers was obtained in muscles irradiated (63 ± 28), or irradiated and notexin injected (13 ± 8), 4 weeks before cell grafts (Fig. 6B-IV, 6B-V, 6C). In the latter case, the number of donor-derived fibers compared to that obtained in muscles either irradiated, or irradiated and notexin injected, 3 days before cell grafts was even more significantly reduced, suggesting that donor satellite cell-mediated regeneration was impaired due to endogenous radiation-resistant satellite cells having partially refilled the niche (supporting information Fig. S4). However, if host muscles are irradiated without any immediate activation of radiation-resistant host satellite cells and grafted 4 weeks later with donor cells, when there are no endogenous satellite cells remaining (Fig. 1C), the niche is no longer able to be refilled either by donor (Fig. 6B-IV, 6C) or endogenous [24] satellite cells.

**Figure 7:**
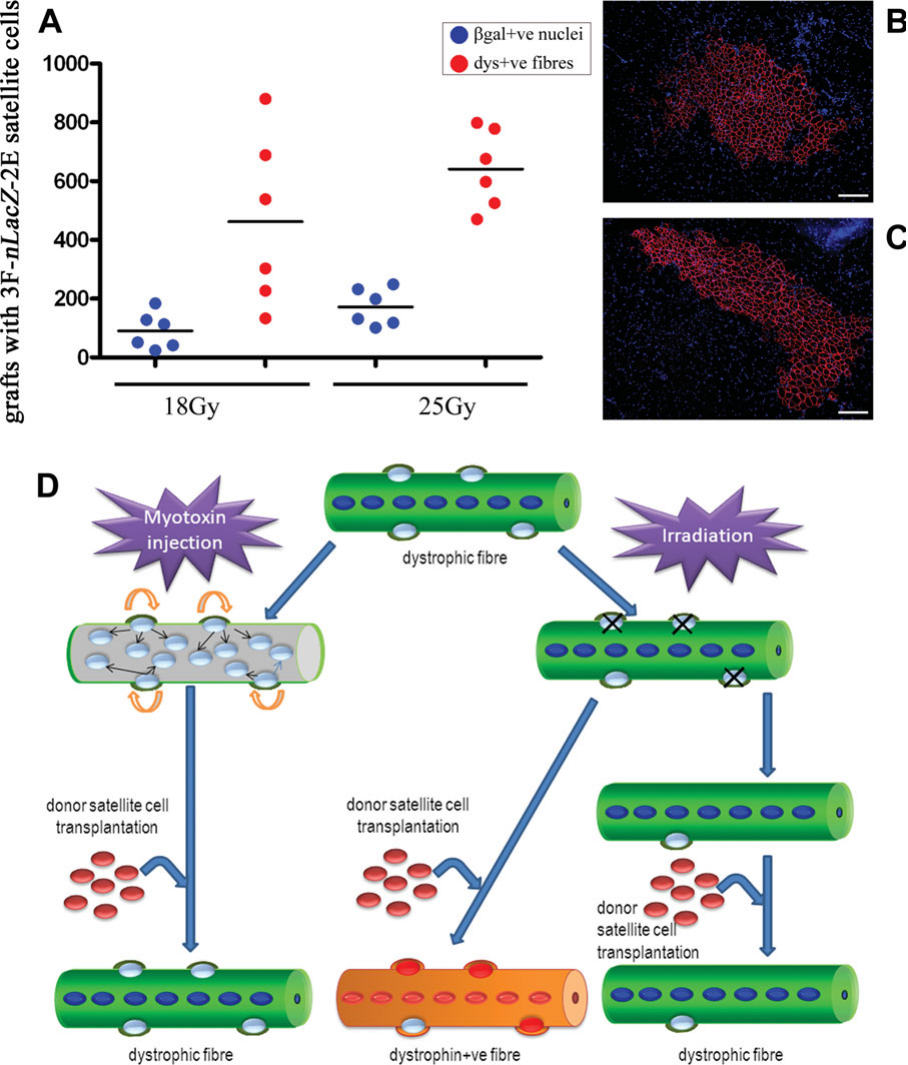
Efficient donor cell engraftment requires not only ablation of endogenous satellite cells but also a functional niche. (**A**) Satellite cells, isolated from 3F-*nLacZ*-2E donor mice (*n* = 2), formed a similar amount of donor-derived fibers in host mice that had their *tibialis anterior* (TA) muscles irradiated with 18 (*n* = 6) or 25 Gy (*n* = 6) immediately before grafting. Graph depicts the amount of dystrophin-positive fibers and donor-derived myonuclei in individual grafts. **(B, C):** Representative images of donor-derived dystrophin-positive fibers (red) in TA muscles that had been irradiated with 18 and 25 Gy, respectively. Nuclei are counterstained with DAPI. Scale bar = 100 μm. The diagram in **(D)** summarizes our model: if the dystrophic fiber is destroyed by myotoxin, endogenous unaffected satellite cells become activated to regenerate the skeletal muscle, and the contribution of donor cells to muscle regeneration is poor. If dystrophic satellite cells are compromised by irradiation, and donor satellite cells are transplanted before the niche becomes dysfunctional following host satellite cell depletion, donor-derived muscle regeneration is efficient.

Similarly, almost no donor-derived regeneration occurred in host muscles that had been irradiated with 25 Gy 3 days before grafting (Fig. 5C). We wished to test the hypothesis that niches irradiated with 25 Gy were still functional immediately (when host satellite cells are still present), but not 3 days, after irradiation (when host satellite cells are absent). We found similar high levels of donor satellite cell engraftment in muscles that were grafted immediately after 18 or 25 Gy irradiation (Fig. 7A–7C), confirming our hypothesis. We conclude that the niche, if not reconstituted shortly after irradiation, becomes dysfunctional (Fig. 7D). Thus, not only impairment of endogenous satellite cells but also retained functionality of the niche is required for efficient donor satellite cell-derived muscle regeneration.

## DISCUSSION

Our work shows the critical importance of the niche for the function of a tissue-specific stem cell. By carefully comparing different methods to modify the host muscle, we investigated the effect of the muscle niche on donor satellite cell-derived muscle regeneration. This was performed in a rigorous systematic, controlled experimental model, in which the genetic background of the host and donor mice, the donor cell preparations, and the time points studied were constant. As hosts, we used *mdx* mice on the nude background [31]. Although it is dystrophin-deficient, skeletal muscles of the *mdx* mouse regenerate satisfactorily and in this respect the *mdx* mouse is not a perfect model of DMD. Nevertheless, *mdx* mice do show progressive degeneration and weakness with age [63]. In addition, we have shown that being on an immunodeficient background exacerbates the muscle pathology: 9-month-old *mdx* nude mice exhibit pathological features characteristic of much older *mdx* mice [37].

We used robust, previously validated markers for muscle fibers and satellite cells of donor origin to carefully quantify donor-derived muscle regeneration [37, 41]. We also compared host muscles that had been injured either on the day of grafting, or up to 3 days beforehand, so that host muscles will contain muscle fibers at different stages of necrosis, satellite cells at different activation and proliferation stages, and different numbers and types of inflammatory cells [53].

Using this experimental system, we did not find significantly more muscle fibers of donor origin in host muscles that had been injured by cardiotoxin, notexin, or cryoinjury, compared to the very small amount of donor muscle found in noninjured host muscles. At first glance, this is in contrast to many published studies in which donor cells were grafted into cryoinjured [38, 40, 64], barium chloride [32, 65], notexin [53, 54], or cardiotoxin-treated host muscles [66, 67]. But few of these studies either quantified the amount of donor muscle formed by grafted cells or compared it to the amount found in noninjured host muscles [40]. Furthermore, different types of cells (muscle precursor cells, primary myoblasts, satellite cells, side population cells, muscle stem cells, and fibro/adipogenic progenitors), different species of donor (mouse and human), and host mice of different pathological (*mdx* and normal) and immunological status (nude, SCID, and C5-/rag2-/gamma chain-) were used, so it is impossible to perform an interstudy comparison. Finally, most studies rarely test in parallel more than one intervention to modify the host environment.

Within injured muscles, host satellite cells are activated [68], migrate toward the site of injury [69], and therefore compete with donor cells. Even when local satellite cells, in addition to muscle fibers, are destroyed by cryoinjury [27], host satellite cells from either the injured or neighboring muscles are able to regenerate freeze-killed muscles [64, 70, 71]. Satellite cells in host muscles that were injured at the time of grafting would be just activating from quiescence [10] and at 3 days after injury would be commencing differentiation [72]. As well as changes in satellite cells, the inflammatory milieu of host muscle and cytokines and growth factor levels change with time after injury: neutrophils peak at 6–24 hours, phagocytic macrophages at 2 days, and nonphagocytic macrophages at 4 days after acute injury (reviewed [73]); in addition, fibro-adipogenic precursors increase in number after acute damage, peaking at 4 days after injury and may produce cytokines that promote satellite cell differentiation [53]. But in our host mouse model, donor satellite cells made similar, small, amounts of muscle when grafted either at the time of cardiotoxin or notexin injection, or up to 3 days later. This suggests that the activation and proliferation status of the host satellite, fibro-adipogenic, or inflammatory cells do not influence the function of grafted satellite cells. As donor satellite cells contribute little to muscle regeneration in nonirradiated environments in which host satellite cells are undamaged [37], we suspect that endogenous satellite cells within their niche outcompete donor satellite cells. The finding that myotoxin-treated muscles that have not been grafted with donor cells regenerate extensively (Fig. 1) lends support to this conjecture.

Donor satellite cells contributed efficiently to muscle regeneration when the host muscle had been irradiated with 18 Gy not only 3 days [37, 41, 52] but also 2 days, 1 day, or even just hours before grafting. This shows that the modulating effect of radiation is extremely rapid, yet lasts for at least 3 days. In contrast, the effect of 25 Gy is markedly different: satellite cells die much more rapidly after 25 than 18 Gy (Fig. 5A) and 25 Gy irradiation of host muscle promotes donor cell-mediated regeneration only immediately, but not 3 days later. In addition, we found no change in the area of inflammation, which is a feature of *mdx* muscles [63], either immediately or 3 days after irradiation (Fig. 1 and supporting information Fig. S1), so we assume that the number of inflammatory cells is not a critical mediator of donor muscle regeneration. Our finding of no changes in cytokine expression that might be responsible for augmenting donor satellite cell engraftment is of interest and argues against the radiation-mediated effect being a globally acting cytokine and suggests that the changes to the host muscle are very local. Our data show that some viable host satellite cells are required to keep the host niche functional: 3 days after 18 Gy irradiation some satellite cells remain and robust donor-derived engraftment occurs, while almost complete ablation of host satellite cells (3 days after 25 Gy irradiation or 4 weeks after 18 Gy irradiation) does not promote engraftment. However, if host satellite cells are not incapacitated by irradiation (e.g., those in nonirradiated muscles or that survive 4.5 or 9 Gy), they outcompete donor cells, impeding donor cell engraftment.

Of interest to an ageing population, aged donor stem cells function very well within an aged environment: host muscle, modified by the optimal radiation regimen, becomes permissive to donor satellite cell-derived muscle regeneration, irrespective of the age of the host muscle or donor satellite cells. This finding adds to a small, but growing, body of literature that disputes the dogma in the field that both the aged muscle niche and satellite cells from aged muscle are regeneration impaired [74].

Ideally, a therapy for muscular dystrophies or sarcopenia would both provide satellite cells to repair damaged muscle, so improving its function, but also reconstitute the satellite cell pool with viable satellite cells able to respond to future myofiber damage. Thus next, we examined self-renewal of donor-derived satellite cells in muscles injured in different ways 3 days prior to grafting (as this was the optimal time point for BaCl2-mediated enhancement of donor cell engraftment), to determine whether there is a correlation between regeneration and self-renewal. It is obvious that irradiated host muscles contained more donor-derived satellite cells than did nontreated, myotoxin-treated host muscles (supporting information Fig. S2). Our finding of the highest incidence of self-renewal and clearly more satellite cells of donor origin in preirradiated host muscles indicates that efficient regeneration and self-renewal are linked. Therefore, when donor satellite cell contribution to muscle regeneration is limited, donor-derived self-renewal is also limited. The fate of donor satellite cells within nonirradiated host muscles remains unclear—they may either die and/or fail to proliferate (as do mouse primary and conditionally immortal myoblasts [75]) and consequently give rise to very few satellite cells or myonuclei of donor origin. Why there is no discernible donor contribution to regenerated muscle fibers in some host muscles (Table 1; Fig. 2 and supporting information Fig. S2) remains unknown.

To further explore the functionality of the niche, we investigated the decrease of endogenous satellite cells and consequent emptying of the satellite niche that occurs as a result of irradiation. As expected in *mdx* mice [24, 76], the loss of satellite cells after irradiation is progressive. Indeed, there is a significant reduction in satellite cell numbers 4 weeks after 18 Gy (Fig. 1 and supporting information Fig. S4) and those that survive are not fully functional [24]. Nevertheless, radiation-resistant satellite cells that are induced to activate by notexin [24, 77] partially refill the satellite cell niche (supporting information Fig. S4). We therefore tested a combination of irradiation and notexin treatment, to determine whether a depleted or partially reconstituted niche affects the efficiency of donor satellite cell engraftment. Irradiation (18 Gy) kills the majority of activated satellite cells in *mdx* nude muscles (Fig. 1, Fig. 5A, supporting information Fig. S4) and notexin activates the radiation-resistant host satellite cells, which are then able to contribute to regenerated fibers and to the satellite cell pool [24].

Our data provide evidence that, as long as it has not been killed or mortally wounded, a host satellite cell in its niche can contribute to muscle regeneration more efficiently than a grafted donor satellite cell. This may be because satellite cells in situ become activated, migrate, or proliferate more rapidly than grafted cells and/or because there are more of them than donor satellite cells. It is possible that some component(s) of the functional host satellite cell niche signal to the incoming donor cells, but this has not been tested.

We illustrate our main findings in Figure 7D. If host satellite cells remain competent (e.g., after myotoxin injury), the necrotic fiber is regenerated by endogenous, rather than donor, satellite cells. In contrast, if host satellite cells are incapacitated by irradiation, donor satellite cells are able to repopulate the niche and contribute efficiently to muscle regeneration. However, if donor satellite cells do not reconstitute the niche promptly, engraftment is extremely poor (Fig. 7D). This implies that all the components of the satellite cell niche—the myofiber, its extracellular matrix, and satellite cells—are required for it to be functional and allow donor satellite cells to contribute to muscle regeneration. There are muscular dystrophies in which the causative genetic defect has either a direct effect on satellite cells, or an indirect effect on them, by affecting the myofiber or basal lamina (reviewed [78]). The irradiated dystrophin-deficient *mdx* mouse muscle, in which muscle regeneration is impaired, models DMD. Our data therefore suggest that donor stem cells would function well within DMD patient muscles.

## CONCLUSIONS

Ideally, incapacitation of the host satellite cell pool, together with retention of sufficient functional niches, would provide an optimal environment for donor cell engraftment of dystrophic muscles. Future work will aim to modify the defective components of the satellite cell niche to optimize stem cell therapies for muscular dystrophies.

## Supplementary material

Supplementary Figure 1 Mdx nude mice, whose hind legs were untreated (n = 3) or irradiated with 18 Gy (n = 5) or 25 Gy (n = 5), had both TA muscles removed either on the same day as irradiation (day 0) (n = 1, 2 and 3 respectively), or 3 days later (n = 1, 2 and 3 respectively). The graph depicts the percentage of CD45+ and CD31+ area found in each case. No significant difference was detected.

Supplementary Figure 2 Self-renewal of donor My5nLacZ/+ satellite cells within host muscles injured in different ways. (a) X-gal staining of whole muscle grafts showed β-gal expressing satellite cells aligned within muscle fibres. Magnifications: 3.2X. (b) Magnified areas highlight donor satellite cell distribution and density along the muscle fibres; donor satellite cells are clearly far more numerous within irradiated host muscles. Magnification: 10X. The incidence of muscles containing donor-derived satellite cells was 5/10 in irradiated, 3/8 in BaCl2-treated, 4/12 in cardiotoxin-treated, 1/8 in notexin-injected muscles and 2/4 in non-treated muscles. We found donor derived satellite cells in ∼36% (15 out of 42) of grafts with no evidence to reject the assumption that different treatments have different efficiencies (Fisher's exact test, p = 0.5627).

Supplementary Figure 3 Heat map display of data collected from cytokine array on irradiated and non-irradiated mdx nude muscles. Proteins extracted from TA muscles of 3 week old mice, irradiated either 3 days before, or on the same day as, analysis were compared to protein extracted from non-treated TA muscles. Analysis was performed on male and female mice (n = 2 in each group).

Supplementary Figure 4 Partial refilling of the niche by radiation-resistant satellite cells. 3 week old mdx nude mice had their TA muscles either irradiated (IRR 4weeks) (n = 6), or notexin injected immediately after irradiation (IRR+NTX 4weeks) (n = 6), or non-treated (n = 6) and removed 4 weeks later. The graph shows quantification of satellite cell number per fibre performed on transverse muscle sections immunostained with Pax7 and laminin antibody. Satellite cell number was significantly lower in treated compared to non-treated muscles, but, there were significantly more satellite cells in IRR+NTX 4weeks than in IRR 4weeks TA muscles. (***p<0.001, * p< 0.05).
