# Supplementary figures and images for: Donor Satellite Cell Engraftment is Significantly Augmented When the Host Niche is Preserved and Endogenous Satellite Cells are Incapacitated
Source: Stem Cells. 2012 Jun 21;30(9):1971–84. doi: 10.1002/stem.1158 (PMC3465801; doi:10.1002/stem.1158)

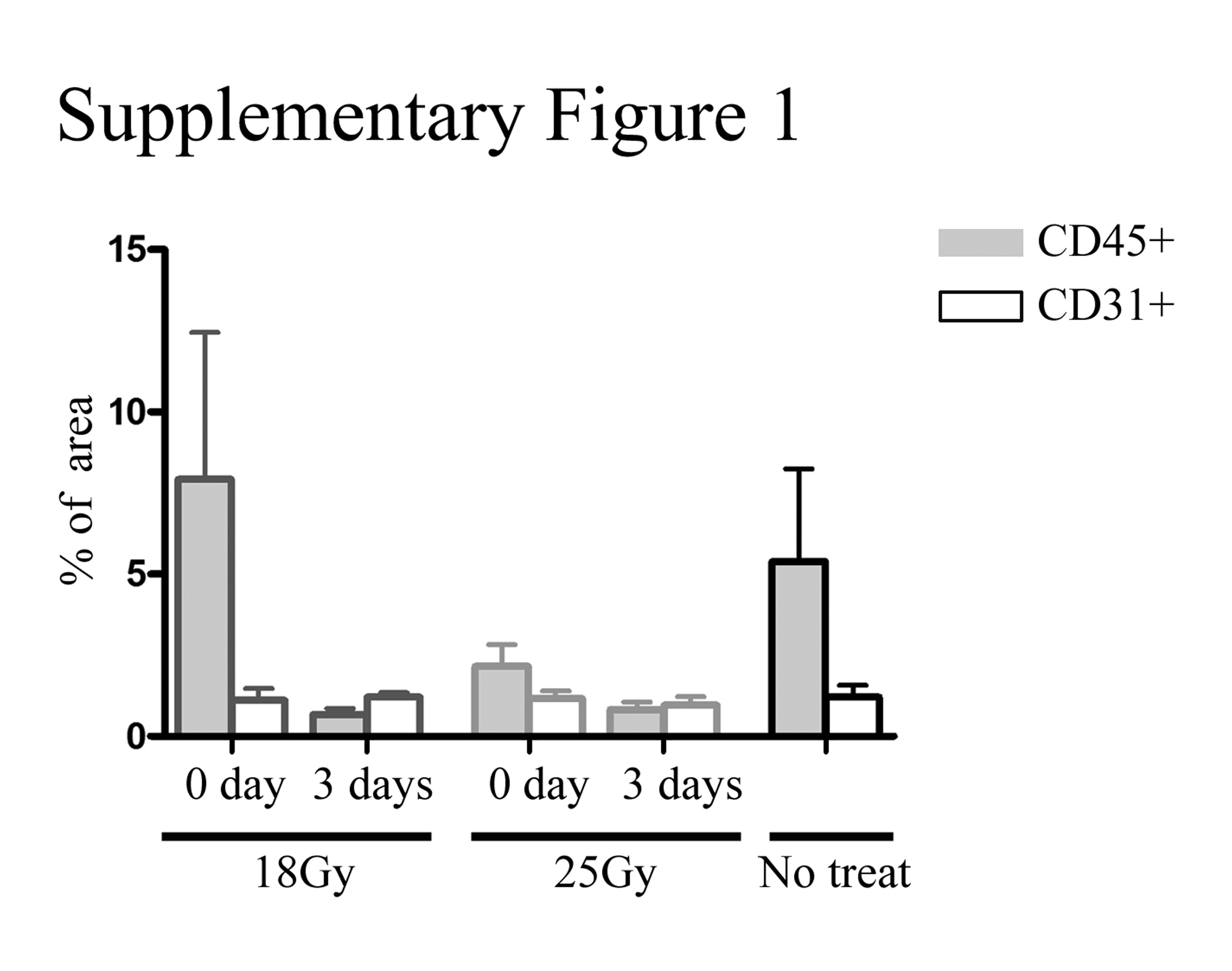

Supplement: Supplementary file 1 [file stem0030-1971-SD1.tif]

Supplementary Figure 2

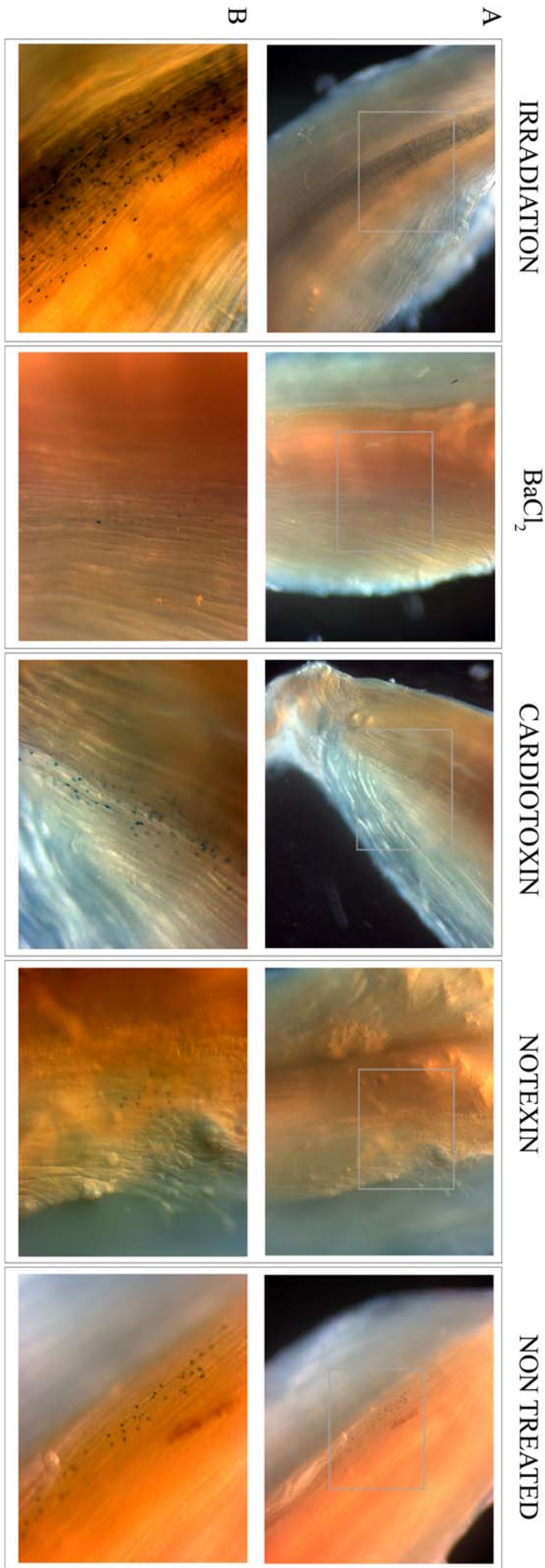

Supplement: Supplementary file 2 [file stem0030-1971-SD2.pdf]

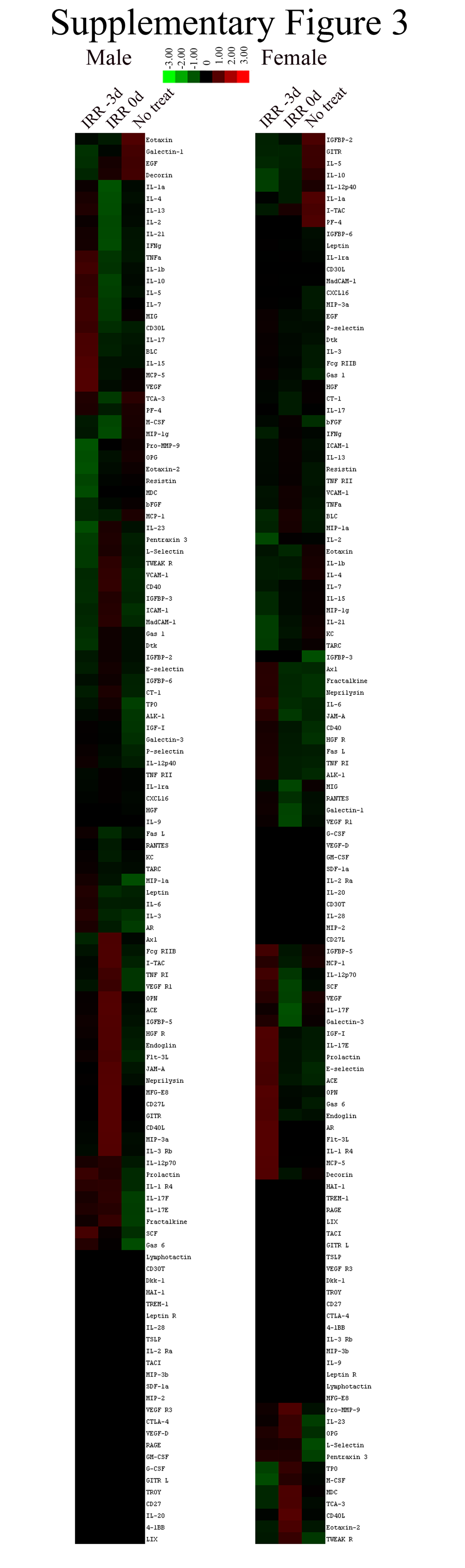

Supplement: Supplementary file 3 [file stem0030-1971-SD3.tif]

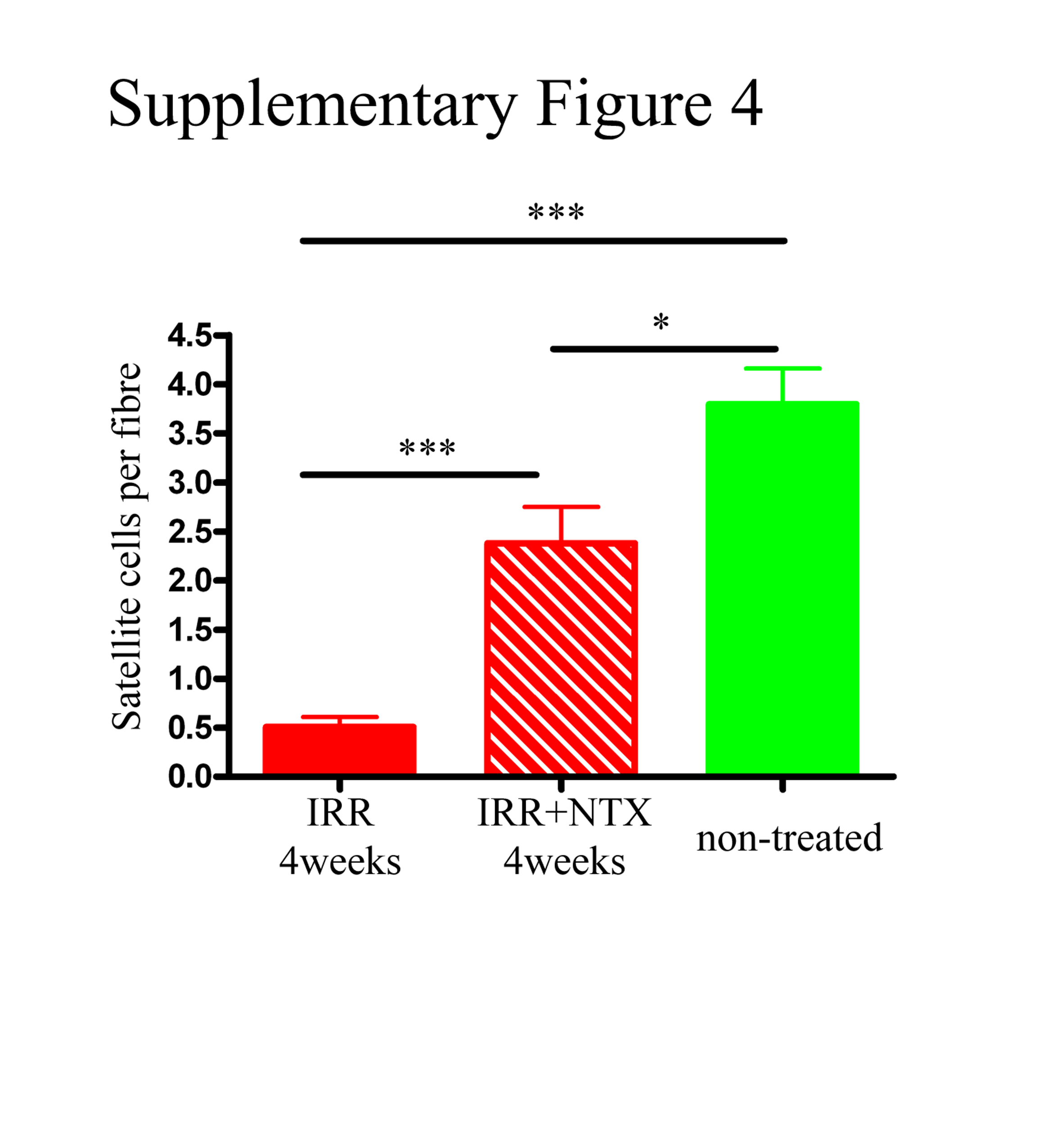

Supplement: Supplementary file 4 [file stem0030-1971-SD4.tif]
